# Mosquito host preferences affect their response to synthetic and natural odour blends

**DOI:** 10.1186/s12936-015-0635-1

**Published:** 2015-03-28

**Authors:** Annette O Busula, Willem Takken, Dorothy E Loy, Beatrice H Hahn, Wolfgang R Mukabana, Niels O Verhulst

**Affiliations:** International Centre of Insect Physiology and Ecology, PO Box 30772-00100 GPO, Nairobi, Kenya; Laboratory of Entomology, Wageningen University, PO Box 8031, 6700 EH, Wageningen, The Netherlands; Departments of Medicine and Microbiology, Perelman School of Medicine, University of Pennsylvania, 3610 Hamilton Walk, Philadelphia, PA 19104-6076 USA; School of Biological Sciences, University of Nairobi, PO Box 30197-00100 GPO, Nairobi, Kenya

**Keywords:** *Anopheles gambiae*, *Anopheles funestus*, *Anopheles arabiensis*, Carbon dioxide, Host seeking, Attraction, Trapping, Monitoring, Anthropophilic

## Abstract

**Background:**

The anthropophilic malaria mosquito *Anopheles gambiae sensu stricto* (hereafter termed *Anopheles gambiae*) primarily takes blood meals from humans, whereas its close sibling *Anopheles arabiensis* is more opportunistic. Previous studies have identified several compounds that play a critical role in the odour-mediated behaviour of *An. gambiae*. This study determined the effect of natural and synthetic odour blends on mosquitoes with different host preferences to better understand the host-seeking behaviour of mosquitoes and the potential of synthetic odour blends for standardized monitoring.

**Methods:**

Odour blends were initially tested for their attractiveness to *An. gambiae* and *An. arabiensis* in a semi-field system with MM-X traps baited with natural and synthetic odours. Natural host odours were collected from humans, cows and chickens. The synthetic odour blends consisted of three or five previously identified compounds released with carbon dioxide. These studies were continued under natural conditions where odour blends were tested outdoors to determine their effect on species with different host preferences.

**Results:**

In the semi-field experiments, human odour attracted significantly higher numbers of both mosquito species. However, *An. arabiensis* was also attracted to cow and chicken odours, which confirms its opportunistic behaviour. A five-component synthetic blend was highly attractive to both mosquito species. In the field, the synthetic odour blend caught significantly more *An. funestus* than traps baited with human odour, while no difference was found for *An. arabiensis*. Catches of *An. arabiensis* and *Culex spp.* contained large numbers of blood-fed mosquitoes, mostly from cows, which indicates that these mosquitoes had fed outdoors.

**Conclusions:**

Different odour baits elicit varying responses among mosquito species. Synthetic odour blends are highly effective for trapping mosquitoes; however, not all mosquitoes respond equally to the same odour blend. Combining fermenting molasses with synthetic blends in a trap represents the most effective tool to catch blood-fed mosquitoes outside houses, which is essential for understanding outdoor malaria transmission.

**Electronic supplementary material:**

The online version of this article (doi:10.1186/s12936-015-0635-1) contains supplementary material, which is available to authorized users.

## Background

The host preference of a mosquito species is an important determinant of its vectorial capacity and mosquito species that are highly anthropophilic are often vectors of important human diseases [1]. The anthropophilic malaria mosquitoes *Anopheles gambiae s.s.* and *Anopheles funestus s.s.*, for example, primarily take blood meals on humans [2] and are two of the most important malaria vectors in Africa [3]. *Anopheles arabiensis*, a close relative of *An. gambiae,* is more opportunistic, feeding on both humans and animals, and is considered a less important malaria vector [1,4]. This difference in host preference is most evident in odour-guided behaviour, where *An. arabiensis* responds more strongly to carbon dioxide (CO_2_) as a general cue to find a host and *An. gambiae* mainly relies on specific human odours [1].

CO_2_ is a major constituent of exhaled air and has been identified as an attractant for many mosquito species including the main vectors of malaria in Africa [5]. Gillies [6] suggested that this compound acts as an activator, initiating flight responses as well as being an attractant. There is strong evidence that CO_2_ acts synergistically with other chemical compounds to attract host-seeking mosquitoes [7-11], which can be used in odour-baited traps in which CO_2_ and synthetic blends that mimic human odour are combined [10,12]. These traps can then be used for monitoring, but can also intercept and reduce the number of malaria mosquitoes entering or leaving houses [9]. A standard synthetic blend (SB) consisting of CO_2_, ammonia, (S)-lactic acid, tetradecanoic acid was tested along with an extended blend to which 3-methyl-1-butanol and butan-1-amine (MB5 blend) was added, and found to be efficient for trapping the malaria mosquito *An. gambiae* in a semi-field setting as well as in two traditional villages in western Kenya [10,13,14]. These attractive blends have been developed for anthropophilic *An. gambiae* mosquitoes [10,11,14-17]. However, less is known about their effect on the host-seeking behaviour of other mosquito species with different host preferences.

In this study, natural host odours and synthetic odour blends were dispensed from mosquito traps to determine the efficacy of synthetic blends for monitoring mosquito species with different host preferences. *Anopheles gambiae* and *An. arabiensis* mosquitoes, which are reported to be anthropophilic and opportunistic respectively [1,4], were simultaneously released in a semi-field system in western Kenya to determine their host-seeking behaviour, either in the presence of CO_2_ alone, or combined with natural odours or the synthetic blends (SB and MB5). In a field trial the efficacy of traps baited with natural odours or a synthetic blend was compared to determine the efficacy of the blend for different species of wild mosquitoes.

## Methods

### Mosquitoes

The semi-field experiments utilized laboratory colonies of the Mbita strain of *An. gambiae sensu stricto* and *An. arabiensis*. Aquatic stages of the mosquitoes were separately reared under ambient atmospheric conditions in screen-walled greenhouses at the Thomas Odhiambo Campus Odhiambo (TOC) of the International Centre of Insect Physiology and Ecology (ICIPE), Mbita, Kenya. Adult mosquitoes were placed in a holding room under ambient conditions with a scotophase of 12:12 h. Female adult mosquitoes were fed three times a week on a human arm [18]. Eggs were laid on moist filter paper and dispensed into plastic trays containing filtered water from Lake Victoria. Newly hatched larvae were transferred into plastic basins and fed on Tetramin® baby fish food (Melle, Germany) three times a day. Collection of pupae until adult emergence is described in Mukabana *et al.* [10]. Female mosquitoes used for semi-field experiments were placed in mosquito netting covered plastic cups [10]. They had no prior access to a blood meal but were fed only on water, provided on wet cotton towels placed on top of mosquito-holding cups during starvation [10]. All semi-field experiments were carried out at night (20:00–06:30 h) inside a 7 × 11 m screenhouse [16]. Two-hundred females of *An. gambiae* and 200 *An. arabiensis* aged three to eight days old were painted with either pink or yellow fluorescent dyes (FTX Series, Astral Pink, Swada, London) ten hours before the experiments, as described before [19]. Mosquitoes were starved for eight hours and simultaneously released at the centre of a screen-walled greenhouse.

### Study sites

Semi-field experiments were conducted between February and April 2013 in a 7×11 m screenhouse constructed on the grounds of the TOC of ICIPE, Kenya (00^0^25^1^S, 34°13^1^E). Field studies were conducted between May and June 2013 at Kigoche village, situated near Ahero town, in the Kano plains of Kisumu County, Kenya (00°34′S, 34°65′ E) [10,20]. The area receives between 1,000 and 1,800 mm of rainfall annually with annual temperature and relative humidity (RH) ranges of 17-32°C and 44-80%, respectively. The long rainy season occurs between March and August while short rains are common in October to November. The main economic activity is rice farming which creates numerous mosquito larval habitats resulting in high malaria transmission. Indigenous goats, cattle, poultry, and sheep are also kept in Kigoche [18]. During the night, domestic animals are tethered outdoors adjacent to houses occupied by humans. Many houses in the area are mud-walled with roofs made of corrugated iron sheets or thatch, or without ceiling. Eaves of most houses are open due to the high daytime temperatures [21]. Previous studies reported that the annual Entomological Inoculation Rate (EIR) was 416 and *An. arabiensis* and *An. funestus s.l.* the main malaria vectors [10,22,23].

### Collection of natural host odours and preparation of CO_2_

Human foot odour previously shown to be moderately attractive to mosquitoes [24] was collected from nylon socks worn by a Kenyan male (age 31) (Additional file 1: Figure S1). The socks were worn for 24 hours before they were used in the experiment [25]. The volunteer did not smoke, use alcohol, spicy food, perfumes and the last shower was without soap [24,26]. Animal odours were collected from the same individual throughout the experiments by wrapping a clean nylon sock above the knee of a cow or around the leg of a chicken for 24 hours (Additional file 1: Figure S1). For the cow odour sample, a piece of cloth was wrapped over the sock to prevent dirt or faeces from contaminating the odour sample. Clean latex gloves were worn to avoid contamination by other odours. Henceforth, human, cow and chicken skin emanations collected on nylon socks will be termed “human odour”, “cow odour” and “chicken odour”, respectively.

Sugar and molasses were used to produce CO_2_ in semi-field and field experiments respectively. Sugar-produced CO_2_ was prepared by mixing 250 g sugar (Mumias Sugar Co Ltd, Kenya), 17.5 g yeast (Angel®Company, China) and 2 L water in 5-L containers which would result in an average CO_2_ production of 242.3 ± 74.1 ml/min [27]. Molasses-produced CO_2_ was obtained by mixing 2 L water, 250 g molasses (Mumias Sugar Co Ltd, Kenya) and 17.5 g dry instant yeast in 5 L containers [18]. Tap water was used during semi-field experiments while all field bioassays were conducted using clean water from Kigoche village. Released CO_2_ was delivered through a 60-cm long silicon tubing (0.5 cm diameter) into individual MM-X traps (American Biophysics, North Kingstown, RI, USA) [18]. The MB5 and the SBs used in the current study were separately prepared following protocols described before [10,13]. Socks containing cow, chicken and human odour, and synthetic blends were separately hooked on a wire ring and hung inside the plume tube of a MM-X trap and always supplied with CO_2_ from either molasses or sugar. Control traps were baited with CO_2_ alone unless indicated specifically. The lower end of the plume tube was suspended 15 cm above ground level [28]. Socks and synthetic blends were placed in glass jars, and stored in a freezer until and between experiments and replaced after four experiments.

### General experimental procedures

All MM-X traps were operated using a 12-V battery. Vaseline pure petroleum jelly was applied on suspension wire bars, electrical cables and CO_2_ tubing to prevent ants from preying on mosquitoes caught in the MM-X traps. To terminate experiments, a plug was inserted into the outer tube of the MM-X trap, the CO_2_ supply was cut off, and the power disconnected [18]. Traps containing mosquitoes were placed in a refrigerator at −4°C for 10 min. Immobilized mosquitoes were collected, counted, and recorded. Traps were cleaned between experiments using 70% ethanol (to remove residual odours). A manual, hand-held aspirator was used to collect untrapped, free-flying mosquitoes from the screenhouse. The sand-filled floor of the greenhouse was moistened daily to enhance survival of mosquitoes. Latex gloves were worn during experiments to avoid contamination with human volatiles or other odorant compounds.

### Attractiveness of natural host odours to laboratory-reared *Anopheles gambiae* and *Anopheles arabiensis*

MM-X traps were placed in all four corners of the screenhouse, and rotated with identical treatments placed at opposite corners of the house. A total of 8 replicates (for a total of 4 nights) were carried out. The treatment combination included: (i) CO_2_*vs* no stimulus; (ii) cow odour + CO_2_*vs* clean sock + CO_2_; (iii) chicken odour + CO_2_*vs* clean sock + CO_2_; and, (iv) human odour + CO_2_*vs* clean sock + CO_2_.

### Attractiveness of natural host odours to *Anopheles gambiae* and *Anopheles arabiensis* by competition

Randomized 4 × 4 Latin square experimental design was adopted. MM-X traps were placed in all four corners of the screenhouse and treatments rotated for 4 consecutive nights. A total of 16 replicates were carried out. The treatment combination included: (i) only CO_2_ and clean sock (control); (ii) cow odour + CO_2_; (iii) chicken odour + CO_2_; and, (iv) human odour + CO_2_.

### Attractiveness of synthetic odour blends to *Anopheles gambiae* and *Anopheles arabiensis*

Randomized 4 × 4 Latin square experimental design was adopted. MM-X traps were placed in all four corners of the screenhouse and treatments rotated for four consecutive nights. A total of 12 replicates were carried out. The treatment combination included: (i) only clean nylon strips without CO_2_ (control); (ii) clean nylon strips + CO_2_; (iii) Simple Blend (SB: NH3 + Lactic acid + C14, [15]) + CO_2_; and, (iv) Mbita blend (MB5: NH3 + Lactic acid + C14 + 3-methyl-1-butanol + Butan-1-amine [13,14]) + CO_2_.

### Response of wild mosquitoes with different host preferences to natural and synthetic odour blends

Five village houses were selected and experiments were carried out from 18.30 to 06.30 h each night. Randomized 5 × 5 Latin square experimental design was adopted. One MM-X trap was placed at each house and treatments rotated for five consecutive nights. A total of 25 replicates were carried out. The treatment combination included a MM-X trap with CO_2_ produced by molasses fermentation and (i) clean sock; (ii) sock with cow odour; (iii) sock with chicken odour; (iv) sock with human odour; and, (v) MB5 blend.

The houses were mud-walled, had open eaves, and corrugated iron sheet roofs and had owner occupants throughout the night sleeping under untreated bed nets. The houses were located at least 25 m apart [29] to exclude the potential interaction of treatments placed in any two adjacent houses. All the baited MM-X traps were hung outside the bedroom window, under the eaves at 15 cm high [17].

### *Anopheles* species identification

Adult mosquitoes were identified morphologically [30] and abdominal status was recorded (Empty (E), blood fed (F), gravid (G)) [31]. Female *An. gambiae s.l.* and *An. funestus s.l.* were preserved in 2-ml Eppendorf tubes containing 80% ethanol and a subset (215 fully blood-fed *An. gambiae s.l.* and 92 unfed *An. funestus s.l.*) was selected for DNA extraction (Qiagen DNeasy kit) and molecular analysis. *Anopheles gambiae* species were identified using a multiplex PCR approach as previously described [32], while *An. funestus* species were determined by PCR amplification, sequencing and phylogenetic sequence analysis of a 380–704 bp fragment of the rDNA gene using primers designed to amplify coding regions flanking the internal transcribed Spacer Region 2 (ITS2) domain [33].

### Blood meal identification and detection of *Plasmodium*

Blood meals were identified using two PCR-based approaches. The first method utilized species-specific primers targeting a fragment (132–680 bp amplicon) of mammalian *cytochrome b (cytb)* [34]. To ensure sensitive detection of mixed blood meals using this method, DNA from each blood fed mosquito was amplified in individual reactions, containing either a human-, cow-, goat-, pig-, or dog-specific forward primer and a universal reverse primer [34]. PCR amplicons were sequenced and subjected to phylogenetic analysis to verify blood meal origin. Second, to ensure sensitive detection of human blood meals, DNA from each mosquito was amplified using primers designed to target the hypervariable D-loop region of ape mitochondrial DNA [35], sequenced, and subjected to phylogenetic analysis.

To screen for the presence of *Plasmodium* parasites in field caught mosquitoes, DNA extracted from whole mosquitoes was subjected to nested PCR targeting a 956 bp *cytb* fragment of the *Plasmodium* mitochondrial genome [36,37]. All PCR reactions used previously reported cycling conditions and the Roche Expand Long Template PCR system.

### Ethical considerations

Scientific and ethical approval of the present study was granted by the Kenya Medical Research Institute (KEMRI/RES/7/3/1). Consent for houses used in the study was obtained from the household heads and the local administration prior to the start of the study.

### Statistical analysis

A generalized linear model (GLM assuming a Binomial distribution with logit link function) was used to investigate the relative attractiveness of each combination of odours tested in the traps in the semi-field and field experiments, expressed as the number of mosquitoes of one species caught in one of the traps divided by the total number of mosquitoes of that species trapped in all traps during each experimental night [38,39]. The effects of treatment, position of trap or house on mosquito catches were fitted in the model and the non-significants dropped. Models were compared by the Pearson Chi-square value divided by the degrees of freedom. Differences between treatments were tested by pair-wise comparisons with least square differences (LSD) correction [40]. Effects were considered significant at P <0.05. All analyses were performed using IBM SPSS statistical software, version 22.

## Results

### Attractiveness of natural host odours to laboratory-reared *Anopheles gambiae* and *Anopheles arabiensis*

**Figure 1:**
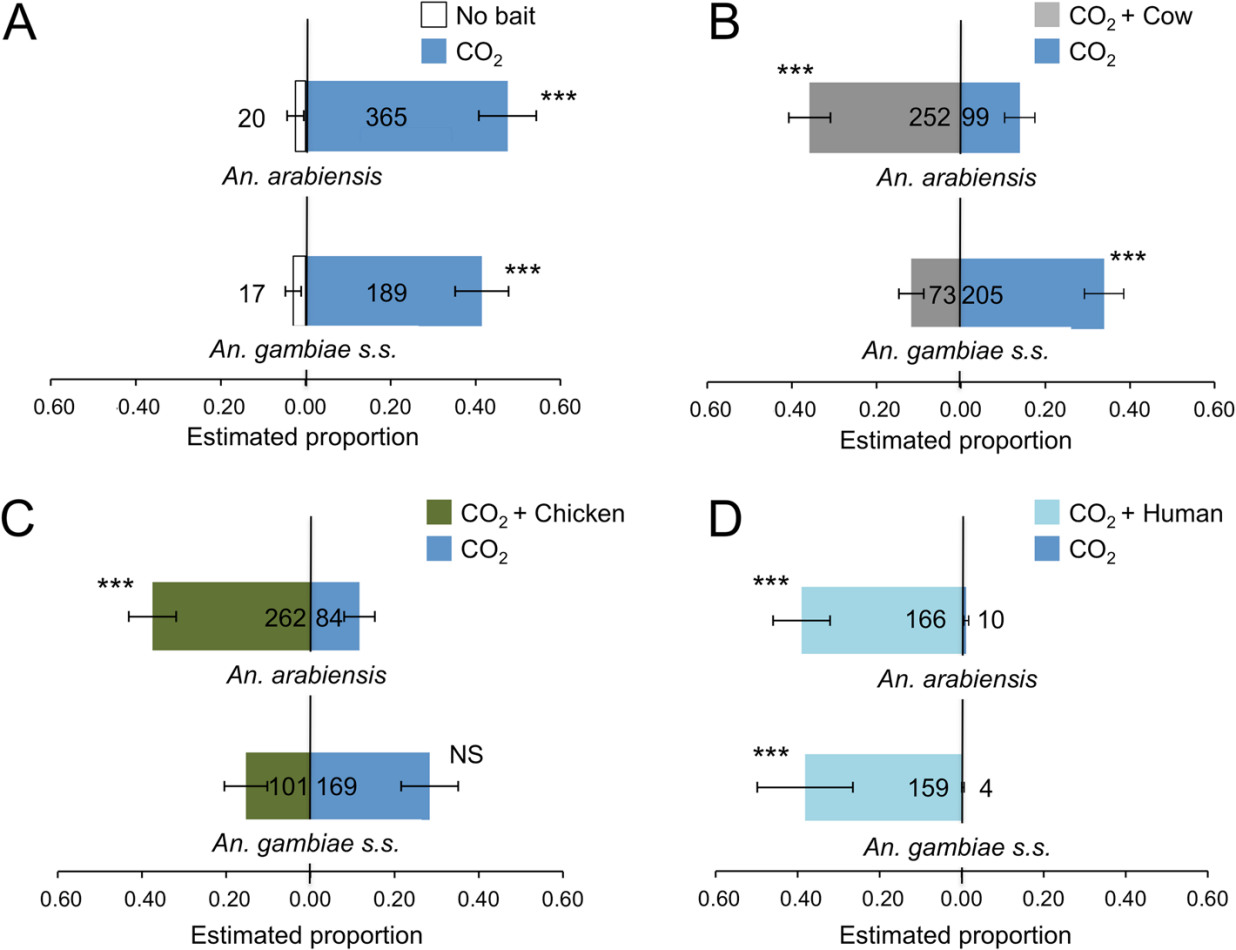
**Screenhouse mosquito catches in dual-choice test with different odour baits.** Estimated mean proportion (GLM) of mosquitoes caught in a screenhouse using MM-X traps with CO_2_ tested *versus* an empty trap **(A)**. Cow **(B)** chicken **C)** and human **(D)** emanations were tested in combination with CO_2_ *versus* a trap with CO_2_ alone. Error bars represent the standard error of the mean; ***: χ^2^-test P < 0.001, NS: χ^2^-test P > 0.05. Numbers in the bars indicate number of mosquitoes caught.

Results from semi-field studies using laboratory-reared mosquitoes showed significant results among MM-X traps baited with different combinations of odours and CO_2_, as follows (P < 0.001, GLM, Figure 1): i) significantly higher number of both *An. gambiae* and *An. arabiensis* in traps baited with CO_2_ than in traps without CO_2_ (Additional file 1: Table S1); ii) significantly lower numbers *An. gambiae* and higher numbers of *An. arabiensis* in traps baited with cow odours compared to traps with CO_2_ alone (Additional file 1: Table S2), iii) significantly higher numbers of *An. arabiensis* in traps baited with chicken odours compared to traps with CO_2_ alone; iv) significantly higher number of both species in traps baited with human odours compared to traps with CO_2_ alone.

### Attractiveness of natural host odours to *Anopheles gambiae* and *Anopheles arabiensis* by competition

**Figure 2:**
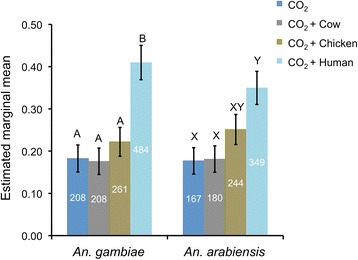
**Competition experiment in a screenhouse with traps baited with natural odours from different host species.** Estimated mean proportion (GLM) of mosquitoes caught in a screenhouse using MM-X traps with CO_2_ only (control), or CO_2_ and cow, chicken or human odours. Error bars represent the standard error of the mean. Numbers in the bars indicate number of mosquitoes caught. For each mosquito species: different letters indicate significant differences between treatments for each mosquito species (P < 0.05, GLM).

Of 3,200 mosquitoes of each species released, 1,161 (36%) *An. gambiae* and 940 *An. arabiensis* (29%) were caught during the 16 experimental nights (GLM, Figure 2). The response of *An. gambiae* to traps baited with human odour was significantly higher than to the other treatments (P <0.05; GLM, Figure 2). The response of *An. arabiensis* was significantly higher to human odour than to cow odour or CO_2_ alone (P <0.001), and close to significant when compared to chicken odour (P = 00.061, GLM, Figure 2, Additional file 1: Table S3).

### Attractiveness of synthetic odour blends to *Anopheles gambiae* and *Anopheles arabiensis*

**Figure 3:**
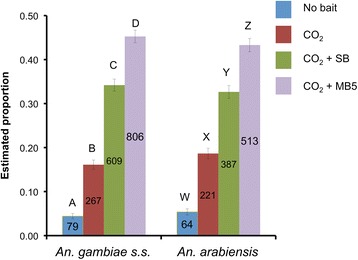
**Screenhouse mosquito catches in traps baited with synthetic blends.** Estimated mean proportion (GLM) of mosquitoes caught in a screenhouse using MM-X traps without (strips only) and with CO_2_ (control) or with CO_2_ plus synthetic blends. Error bars represent the standard error of the mean. Numbers in the bars indicate number of mosquitoes caught. For each mosquito species: different letters indicate significant differences between treatments (P < 0.05, GLM).

The attractiveness of all treatments was significantly different for both mosquito species, (P <0.001, GLM, Figure 3, Additional file 1: Table S4). The trap without CO_2_ was least attractive to mosquitoes, followed by the traps baited with CO_2_ alone, and then CO_2_ + SB. Traps baited with CO_2_ plus the MB5 blend were the most attractive to mosquitoes (GLM, Figure 3).

### Response of wild mosquitoes with different host preferences to natural and synthetic odour blends

A total of 6,057 wild mosquitoes were caught outdoors in Kigoche village over a period of 25 nights between May and June 2013. Of the 6,057 mosquitoes, 6% (n = 367) were males and 94% (n = 5,690) were females (Additional file 1: Table S5). Out of the 5,690 female mosquitoes trapped, 9% (n = 535) were blood fed (F) and none was gravid (HG, G).

**Figure 4:**
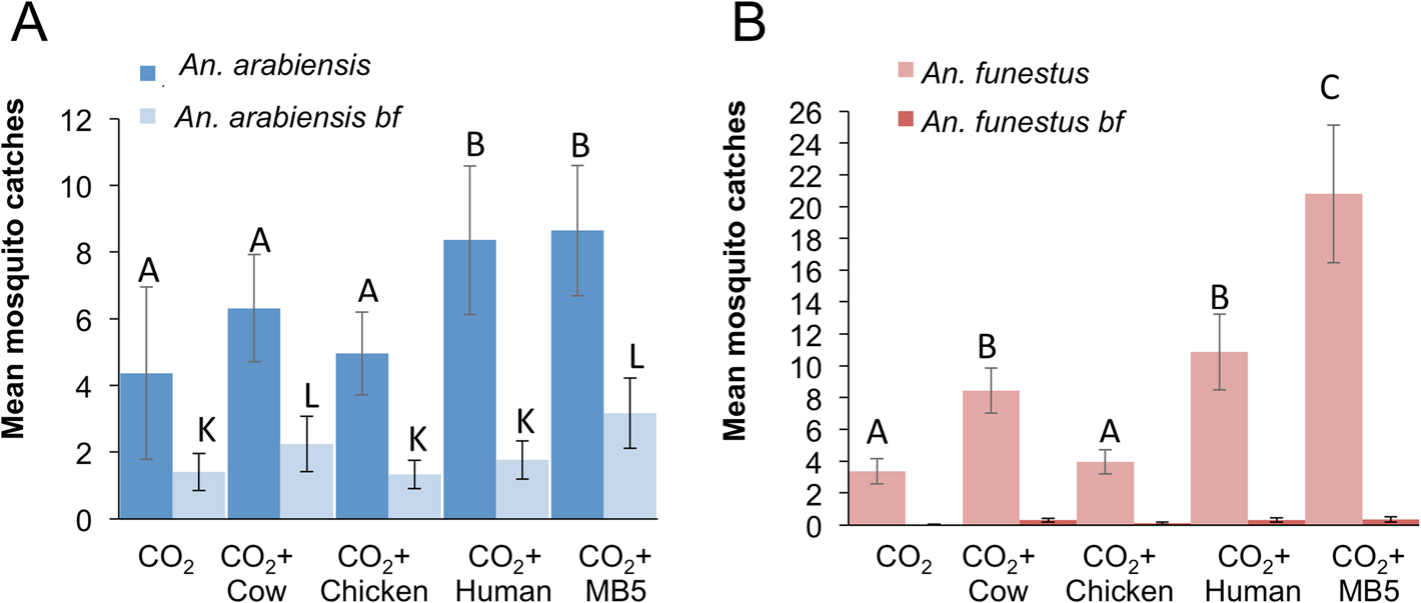
**Mosquito catches in traps baited with natural and synthetic odours in a field set-up.** Estimated mean proportion (GLM) of wild unfed or blood-fed mosquitoes caught outdoors. **A)** *An. arabiensis*, **B)** *An. funestus* s.l. caught outdoors using MM-X traps with CO_2_ or CO_2_ and treatments. Numbers in the bars indicate number of mosquitoes caught during 25 experimental nights. For each mosquito species: different letters indicate significant differences between treatments (P < 0.05, GLM).

For the ‘unfed’ mosquitoes, 16% (n = 816) were *An. arabiensis,* 23% (n = 1,186) *An. funestus*, 35% (n = 1,803) *Culex spp.*, 20% *Mansonia spp.* (1,028) and 6% (n = 322) were other mosquito species. There was no significant difference in numbers of *An. arabiensis* caught in traps baited with CO_2_ alone and traps baited with cow or chicken odours (P = 00.273, P = 00.703, respectively, GLM, Figure 4A, Additional file 1: Tables S6 and S7). Human and MB5-baited traps attracted equal numbers of *An. arabiensis* (P = 00.887) and the catches were significantly higher than those of CO_2_, cow or chicken-baited traps (GLM, Figure 4A, Additional file 1: Tables S6,S7). For unfed *An. funestus*, CO_2_ and chicken odour were least attractive (P = 00.696, GLM Figure 4B, Additional file 1: Tables S6, S7). Cow or human odours were more attractive to *An. funestus* (P = 00.292) with higher catches than CO_2_ (P = 00.007) or chicken (P = 00.020) but lower than the MB5 blend which was most attractive to *An. funestus* (P < 0.001, GLM Figure 4B, Additional file 1: Tables S6 and S7). Compared to the response to CO_2_ alone, the *Culex spp.* did not show any enhanced attraction to the traps when natural odours or the synthetic blend were added (P > 0.05, GLM, Additional file 1: Tables S6, S7). *Mansoni spp.* were more attracted to cow odour and the MB5 blend compared to traps baited with CO_2_ alone (P = 00.010 and P = 00.007 respectively, GLM, Additional file 1: Tables S6 and S7).

Carbon dioxide production by fermenting molasses resulted in a high percentage of blood fed mosquitoes [18]. A total of 535 out of 6,057 collected females were blood fed: 45% were *An. arabiensis*, 5% were *An. funestus*, 38% were *Culex spp.*, 8% *Mansonia spp.* and 3% were other mosquito species (Figure 4). There was a significantly higher number of blood fed *An. arabiensis* caught by MB5 compared to CO_2_, chicken or man (P < 0.036) but not compared to cow odour baited traps (P = 00.142, GLM, Figure 4A, Additional file 1: Tables S7 and S8). Blood-fed *An. funestus* were caught more often in traps baited with cow odour, human odour and the MB5 blend than traps baited with CO_2_ alone although numbers were relatively low for a GLM analysis (P < 0.021, GLM, Figure 4B, Additional file 1: Tables S7 and S8). No significant differences were found between *Culex spp.* and *Mansonia spp.* trapped with the different treatments (P > 0.05, GLM, Additional file 1: Tables S7 and S8).

### Molecular characterization of mosquitoes caught in field settings

To confirm the species origin of wild-caught mosquitoes, 215/240 (from 25 samples the ethanol evaporated) fully blood-fed *An. gambiae s.l.* and a subset of 92 unfed *An. funestus s.l* were subjected to mitochondrial DNA analysis. In concordance with previous studies [18,41], all *An. gambiae s.l.* were identified as *An. arabiensis* except for one that could not be typed due to insufficient material. Similarly, all *An. funestus s.l.* analysed were identified as *An. funestus s.s*. Analysis of the same mosquitoes for the presence of *Plasmodium* (*cytb)* sequences revealed that two *An. funestus* were positive for *Plasmodium falciparum* and one *An. funestus* was positive for *Plasmodium malariae* (Additional file 1: Figure S3)*.* None of the *An. arabiensis* was *Plasmodium* sequence positive. Blood meal analysis revealed that the vast majority of blood fed *An. arabiensis* contained cow blood (86%), with a small minority also containing human blood (2%) as determined by sequence analysis of mitochondrial PCR amplicons (Table 1, Additional file 1: Figure S2). Additionally, one human, one caprid and one canine blood meal were identified. Twenty-nine *An. arabiensis* did not yield blood meal PCR amplicons by either method.

**Table 1:** **Blood meal identification in field caught** ***An. arabiensis***

**Blood meal origin**	**No. (percent)**
*Single species blood meal*	
Cow	180 (83.3)
Caprid	1 (0.5)
Dog	1 (0.5)
Human	1 (0.5)
*Multiple species blood meal*	
Cow and human	4 (1.8)
*Blood meal undetectable*	29 (13.4)

## Discussion

CO_2_ has been identified as an attractant for many mosquito species [5,6,27,42,43]. The semi-field experiments presented here confirm that CO_2_ is an important cue for both *An. gambiae s.s.* and *An. arabiensis* [5,6] and that including CO_2_ in monitoring traps increases their efficacy (Figure 1). Adding host odours to CO_2_ increased trap catches for *An. arabiensis*, but results were less clear-cut for *An. gambiae*. Human odour was highly attractive to both species (Figures 1 and 2) and although this has been reported previously for *An. gambiae* in both field and laboratory studies [1,25,44], only a few studies have reported *An. arabiensis* to be more attracted to human compared to cow odour [1,45-47]. The results show that *An. arabiensis* is opportunistic in nature. Moreover, human odour appeared to be more important than cow or chicken odour in the attractiveness to female *An. arabiensis*, although individual differences in attractiveness could have played a role.

Interestingly, in some of the semi-field experiments, adding cow or chicken odour to traps baited with CO_2_ decreased the number of *An. gambiae s.s.* caught (Figure 1). This effect has been reported before when CO_2_ was added to cow odour in an olfactometer, however, when only cow odour without CO_2_ was present, the inhibiting effect was not observed [25]. A field study by Costantini *et al.* [48] also indicated an aversion of *An. gambiae s.s.* to cattle odour when using odour-baited entry traps. These studies and the results presented here further confirm the anthropophilic nature of this mosquito species and the importance of both human odour and CO_2_ in its host-seeking behaviour.

The MB5 blend has proven to be an effective synthetic blend for monitoring malaria mosquitoes [13,14]. However, it was not clear from previous studies whether this blend would attract different species equally, and whether the host preference of these species would affect their preference to these blends. Results of semi-field experiments show that *An. gambiae s.s.* and *An. arabiensis* host preferences do not influence their response to the MB5 blend, which is attractive for both species. Nevertheless, a substantial proportion of the mosquitoes did not get trapped in the traps baited with either natural or synthetic odours and it is unclear if these mosquitoes escaped the screenhouse, were not host seeking, were influenced by the weather or were not trapped for other reasons. Field experiments also revealed a clear difference in response between the two important malaria vectors *An. funestus* and *An. arabiensis*. Although human odour and the MB5 blend attracted equal numbers of *An. arabiensis*, the synthetic blend attracted significantly more *An. funestus s.s.* than traps baited with human odour. Particular odour baits selected for monitoring purposes will therefore affect both the number of mosquitoes and the ratio between the species collected. The advantage of using the MB5 blend for monitoring is that it is standardized, highly effective (Figure 4) and long lasting (Mweresa, pers. comm.).

No *Plasmodium* was detected in the *An. arabiensis* mosquitoes analysed; however, 3.3% of the *An. funestus s.s.* tested were *Plasmodium* positive. This result may be explained by the zoophilic nature of *An. arabiensis* and more anthropophilic behaviour of *An. funestus s.s.*

Molecular analyses of blood-fed *An. arabiensis* females indicated that 87% of the blood meals were of cow origin and only 2% of human origin. Since traps were hung outside, this result may reflect host availability rather than host preference [49]. True host preference is better evaluated using choice tests [1] as performed in the semi-field experiments; however, host choice will largely depend on the host availability in the field [1]. A previous study by Mweresa *et al.* [18] showed that a trap with fermenting molasses, rather than fermenting sugar, significantly increased the number of blood-fed mosquitoes caught compared to the number of unfed mosquitoes. The blood meal results presented here show that the use of fermenting molasses in a trap can catch mosquitoes that have fed outdoors, since most of the blood meals were from cows and typically cattle are kept outside human habitations. This result indicates that molasses-fermenting traps are very suitable for monitoring outdoor mosquitoes and thereby outdoor transmission.

In the last decade, indoor residual spraying (IRS) and the use of long lasting insecticidal nets (LLINs) have reduced indoor mosquito populations and thereby malaria transmission [50-53]. In areas where indoor transmission has been reduced substantially through the use of LLINs and IRS, the control of outdoor malaria has become more important and there is a need, therefore, for effective tools to monitor and reduce outdoor transmission. Outdoor odour-baited traps have become increasingly efficient for catching host-seeking mosquitoes. Nonetheless, they catch few or no blood-fed mosquitoes [10,12] and methods that permit the reliable and consistent trapping of blood-fed mosquitoes outdoors are not available. The combination of fermenting molasses with selected odour baits represents an important new tool for understanding outdoor mosquito behaviour, which will be of utility to measure, and possibly even reduce, outdoor transmission. To eliminate malaria, targeting outdoor vectors will be essential and odour-baited traps that target both host seeking and blood fed mosquitoes could become an important tool.

Odour baits, including synthetic blends, are biased in their capture efficacy, and in addition, the traps themselves may also bias mosquito catches and the odours to which mosquitoes respond [45]. These are important consideration when monitoring or mass trapping mosquitoes, however, the use of a synthetic odour blend as an attractant in traps remains a very effective and standardized method for mosquito monitoring and possibly reduction.

## Additional file

Additional file 1:
**Collection of host odours from legs of (A) a male human, (B) cow, and (C) from chicken.** **Figure S2.** Blood meal analysis of wild caught *An. arabiensis.* **Figure S3.** Identification of *Plasmodium falciparum* and *Plasmodium malariae* in wild caught mosquitoes. **Table S1.** Mean (±SE) of mosquitoes caught in a screenhouse using MM-X traps with A) without CO_2_, B) cow, C) chicken and D) human odours. **Table S2.** Mean (±SE) of mosquitoes caught in a screenhouse using MM-X traps baited with natural host odours. **Table S3.** P-values of pair-wise comparisons (GLM) after LSD correction, based on proportions of number of mosquitoes caught in a screenhouse by use of natural host odours. The mean difference is significant at the 0.05 level. **Table S4.** Mean (±SE) of mosquitoes caught in a screenhouse using MM-X traps baited with synthetic blends.** Table S5.** Mean (±SE) of wild male mosquitoes caught outdoors using MM-X traps baited with natural host or synthetic odour blends. **Table S6.** Mean (±SE) and standard deviation (SD) of wild non-fed female mosquitoes caught outdoors using MM-X traps baited with natural or synthetic odour blends. **Table S7.** Pair-wise comparisons of P values (GLM) based on proportions of wild mosquitoes caught in MM-X traps baited with natural and synthetic odour blends. The mean difference is significant at the 0.05 level. **Table S8.** Mean (±SE) of wild blood-fed mosquitoes caught outdoors using MM-X traps baited with natural and synthetic odour blends.
