# Supplementary material for: Mosquito host preferences affect their response to synthetic and natural odour blends
Source: Malar J. 2015 Mar 28;14:133. doi: 10.1186/s12936-015-0635-1 (PMC4381365; doi:10.1186/s12936-015-0635-1)
Supplement: Additional file 1: — Collection of host odours from legs of (A) a male human, (B) cow, and (C) from chicken. Figure S2. Blood meal analysis of wild caught An. arabiensis. Figure S3. Identification of Plasmodium falciparum and Plasmodium malariae in wild caught mosquitoes. Table S1. Mean (±SE) of mosquitoes caught in a screenhouse using MM-X traps with A) without CO2, B) cow, C) chicken and D) human odours. Table S2. Mean (±SE) of mosquitoes caught in a screenhouse using MM-X traps baited with natural host odours. Table S3. P-values of pair-wise comparisons (GLM) after LSD correction, based on proportions of number of mosquitoes caught in a screenhouse by use of natural host odours. The mean difference is significant at the 0.05 level. Table S4. Mean (±SE) of mosquitoes caught in a screenhouse using MM-X traps baited with synthetic blends. Table S5. Mean (±SE) of wild male mosquitoes caught outdoors using MM-X traps baited with natural host or synthetic odour blends. Table S6. Mean (±SE) and standard deviation (SD) of wild non-fed female mosquitoes caught outdoors using MM-X traps baited with natural or synthetic odour blends. Table S7. Pair-wise comparisons of P values (GLM) based on proportions of wild mosquitoes caught in MM-X traps baited with natural and synthetic odour blends. The mean difference is significant at the 0.05 level. Table S8. Mean (±SE) of wild blood-fed mosquitoes caught outdoors using MM-X traps baited with natural and synthetic odour blends. [file 12936_2015_635_MOESM1_ESM.docx]

**Additional Figure 1** **Collection of host odours from legs of (A) a male human, (B) cow, and (C) from chicken**


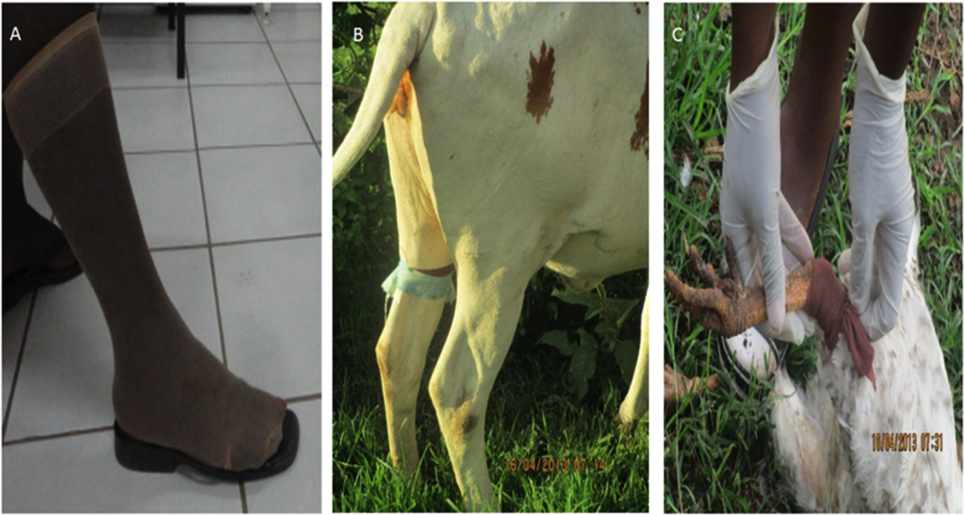


**Additional Figure 2 Blood meal analysis of wild caught *An. arabiensis***

DNA was extracted from wild-caught *An. arabiensis* and used to amplify (A) cytochrome B (*cytb)* and (B) hypervariable D-loop sequences using primers specific for mammalian mitochondrial sequences. (A) Phylogenetic tree of cytochrome B (*cytb*) gene (305 bp) sequences identifying blood meals of dog, sheep/goat, cow and human origins. Seven distinct haplotypes (A-G) are shown in relation to reference sequences for *Bos taurus* (GenBank accession numbers AB090987 and DQ186224), *Homo sapiens* (AY495285 and KM102057), *Hemitragus jayakari* (AY846791), *Pseudois nayaur* (JX101652), *Capra hircus* (D84201), and *Canis lupus familiaris* (KJ637145 and NC_002008). (B), Phylogenetic tree of mitochondrial D-loop (313 bp) sequences, identifying four human blood meal haplotypes (1-4) in *An. arabiensis*. Reference sequences for *Pan troglodytes verus* (KJ606392.1)*, Pan troglodytes troglodytes* (AJ851169.1)*, Pan troglodytes schweinfurthii* (JQ812125.1)*,* and *Homo sapiens* (KC005303.1) are shown. The number of *An. arabiensis* containing the respective blood meal haplotype is indicated in parenthesis. Both trees were inferred using maximum likelihood methods [1] in Geneious version 8.0.4 [2]. Numbers above nodes indicate bootstrap values (> 70 %) and the scale bar represents 5 nucleotide substitutions.

**Additional Figure 3 Identification of *Plasmodium falciparum* and *Plasmodium malariae* in wild caught mosquitoes**

The phylogenetic positions of *Plasmodium* cytochrome B (*cytb)* gene (689 bp) sequences amplified from three wild-caught *An. funestus* mosquitoes (sample numbers 2U25, 2U49, and 2U87) are shown in relation to reference sequences for *Plasmodium falciparum* (AY282973.1)*, Plasmodium malariae* (AB354570.1)*, Plasmodium vivax* (KF591834.1)*,* and *Plasmodium ovale* (AB182497.1). The tree was inferred using maximum likelihood methods [1] in Geneious version 8.0.4 [2]. Numbers above nodes indicate bootstrap values (> 70 %) and the scale bar represents 5 nucleotide substitutions.

**Additional Table 1** **Mean (±SE) of mosquitoes caught in a screenhouse using MM-X traps with A) without CO_2_, B) cow, C) chicken and D) human odours**

N=number of trapping nights


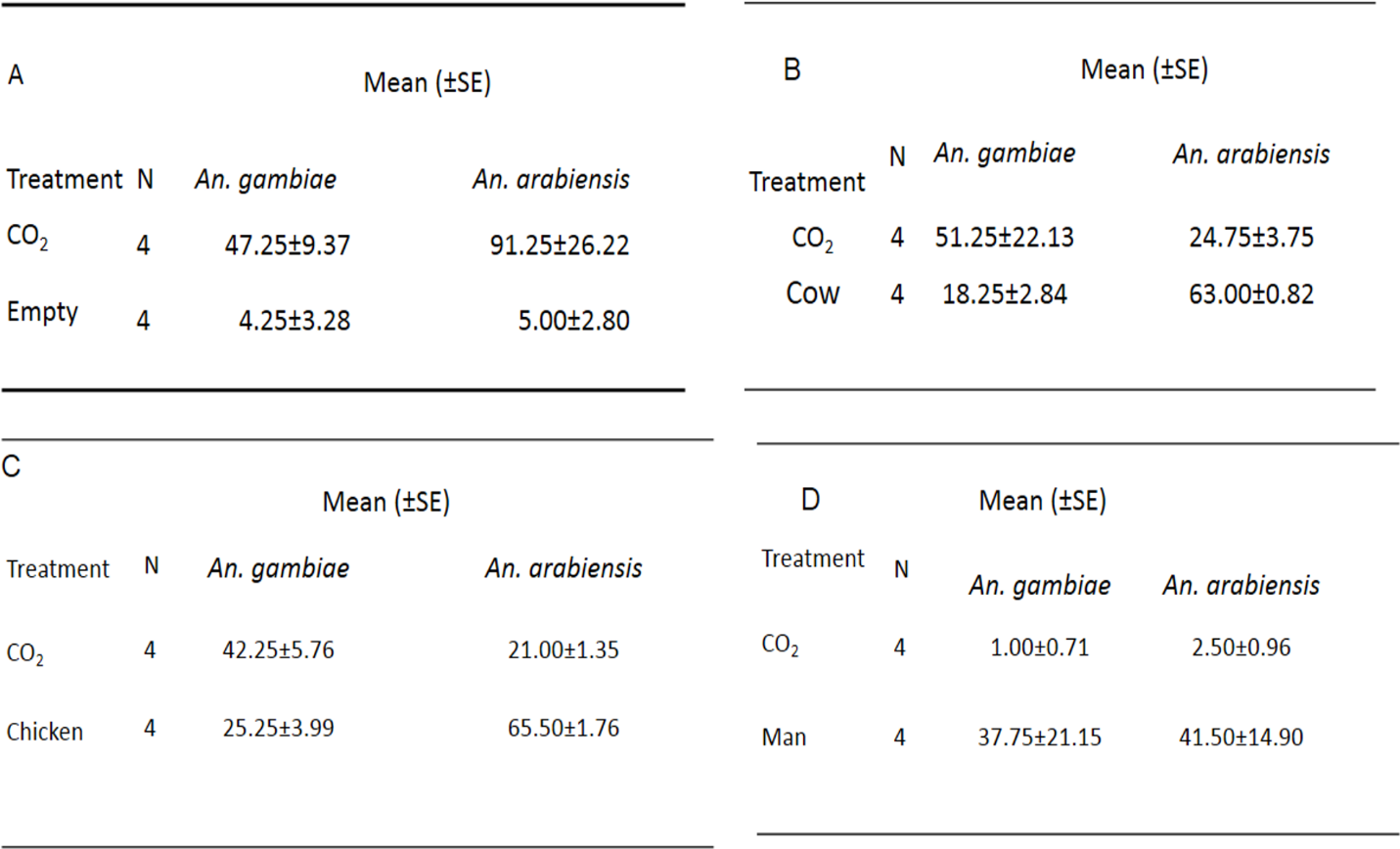


**Additional Table 2** **Mean (±SE) of mosquitoes caught in a screenhouse using MM-X traps baited with natural host odours**

N=number of trapping nights

| Treatment | N | Mean (±SE) | |
| --- | --- | --- | --- |
|  |  | *An. gambiae* | *An. arabiensis* |
| Control (CO_2_ Only) | 16 | 13.00±2.73 | 10.44±1.62 |
| Cow | 16 | 13.00±2.55 | 11.25±2.05 |
| Chicken | 16 | 16.31±2.31 | 15.25±2.99 |
| Human | 16 | 30.25±5.60 | 21.81±3.82 |

**Additional Table 3** **P-values of pair-wise comparisons (GLM) after LSD correction, based on proportions of number of mosquitoes caught in a screenhouse by use of natural host odours**

The mean difference is significant at the 0.05 level

| Treatment | Comparison host odour+CO_2_ | P values  *An. gambiae* | P values  *An. arabiensis* |
| --- | --- | --- | --- |
| Chicken | Control | 0.400 | 0.116 |
|  | Cow | 0.319 | 0.137 |
|  | Human | <0.001 | 0.061 |
| Control | Chicken | 0.400 | 0.116 |
|  | Cow | 0.882 | 0.924 |
|  | Human | 0.000 | 0.001 |
| Cow | Chicken | 0.319 | 0.137 |
|  | Control | 0.882 | 0.924 |
|  | Human | <0.001 | 0.001 |
| Human | Chicken | <0.001 | 0.061 |
|  | Control | <0.001 | 0.001 |
|  | Cow | <0.001 | 0.001 |

**Additional Table 4 Mean (±SE) of mosquitoes caught in a screenhouse using MM-X traps baited with synthetic blends**

N=number of trapping nights

| Treatment | N | Mean (±SE) | |
| --- | --- | --- | --- |
|  |  | *An. gambiae* | *An. arabiensis* |
| Control strips without CO_2_ | 12 | 6.58±1.196 | 5.33±1.05 |
| CO_2_ | 12 | 23.92±1.43 | 18.42±1.12 |
| SB | 12 | 50.75±2.43 | 32.25±1.95 |
| MB5 | 12 | 67.17±2.50 | 42.75±2.26 |

**Additional Table 5 Mean (±SE) of wild male mosquitoes caught outdoors using MM-X traps baited with natural host or synthetic odour blends**

N=number of trapping nights

| Treatment | N | *An. gambiae* s.l | *An. funestus* | *Culex spp.* | *Mansonia spp.* | *An. ziemmani* | *An. coustani* |
| --- | --- | --- | --- | --- | --- | --- | --- |
| Control | 25 | 0.48±0.15 | 0.52±0.24 | 0.24±0.15 | 0.40±0.30 | 0 | 0 |
| Cow | 25 | 0.88±0.32 | 0.68±0.21 | 0.80±0.28 | 0.44±0.25 | 0 | 0.08±0.08 |
| Chicken | 25 | 0.96±0.29 | 0.92±1.73 | 0.52±1.05 | 2.40±2.28 | 0.08±0.06 | 0.24±0.24 |
| Human | 25 | 0.40±0.15 | 0.20±0.10 | 0.40±0.14 | 0.56±0.232 | 0 | 0.08±0.08 |
| MB5 | 25 | 1.32±0.44 | 0.76±0.23 | 0.44±0.22 | 0.68±0.44 | 0.02±0.01 | 0.11±0.06 |

**Additional Table 6 Mean (±SE) and standard deviation (SD) of wild non-fed female mosquitoes caught outdoors using MM-X traps baited with natural or synthetic odour blends**

N=number of trapping nights

|  |  | *An. arabiensis* | *An. funestus* | *Culex spp.* | *Mansonia spp.* | *An. ziemmani* | *An. coustani* | Unidentified |
| --- | --- | --- | --- | --- | --- | --- | --- | --- |
| Control | 25 | 4.36±2.59 | 3.36±0.80 | 13.4±8.12 | 4.80±1.63 | 0.04±0.04 | 0.92±0.65 | 0.16±0.10 |
| Cow | 25 | 6.32±1.61 | 8.44±1.42 | 14.00±2.87 | 10.92±2.77 | 0.08±0.06 | 1.60±0.43 | 0.44±0.21 |
| Chicken | 25 | 4.96±1.25 | 3.96±0.77 | 15.12±3.39 | 7.04±1.49 | 0.60±0.29 | 1.56±0.52 | 0.29±0.13 |
| Human | 25 | 8.36±2.23 | 10.88±2.39 | 16.24±3.56 | 7.92±1.60 | 0.56±0.40 | 2.32±0.57 | 0.44±0.327 |
| MB5 | 25 | 8.64±1.96 | 20.80±4.33 | 13.36±2.72 | 10.44±2.16 | 0.04±0.04 | 3.08±1.27 | 0.76±0.28 |

**Additional Table 7 Pair-wise comparisons of P values (GLM) based on proportions of wild mosquitoes caught in MM-X traps baited with natural and synthetic odour blends**

The mean difference is significant at the 0.05 level

| Treatment | | 1. Non blood-fed mosquitoes | | | | 1. Blood-fed mosquitoes | | | | | | |  |  |
| --- | --- | --- | --- | --- | --- | --- | --- | --- | --- | --- | --- | --- | --- | --- |
|  |  | *An. gambiae* s.l | *An. funestus* | *Culex spp.* | *Mansonia spp.* | | *An.*  *gambiae* s.l | | *An. funestus* | | *Culex spp.* | *Mansonia spp.* | |  |
| Chicken | CO_2_ | 0.703 | 0.696 | 0.675 | 0.176 | 0.945 | | 0.370 | | 0.120 | | 0.826 | | |
|  | cow | 0.423 | 0.020 | 0.787 | 0.172 | 0.072 | | 0.141 | | 0.341 | | 0.525 | | |
|  | human | 0.059 | 0.001 | 0.792 | 0.948 | 0.242 | | 0.141 | | 0.445 | | 0.705 | | |
|  | MB5 | 0.042 | <0.001 | 0.668 | 0.124 | 0.002 | | 0.085 | | 0.252 | | 0.937 | | |
| Control | chicken | 0.703 | 0.696 | 0.675 | 0.176 | 0.945 | | 0.370 | | 0.120 | | 0.826 | | |
|  | cow | 0.237 | 0.007 | 0.882 | 0.01 | 0.059 | | 0.021 | | 0.549 | | 0.673 | | |
|  | human | 0.023 | <0.001 | 0.495 | 0.145 | 0.212 | | 0.021 | | 0.431 | | 0.875 | | |
|  | MB5 | 0.016 | <0.001 | 0.992 | 0.007 | 0.001 | | 0.010 | | 0.684 | | 0.890 | | |
| Cow | chicken | 0.423 | 0.020 | 0.787 | 0.172 | 0.072 | | 0.141 | | 0.341 | | 0.525 | | |
|  | CO_2_ | 0.237 | 0.007 | 0.882 | 0.01 | 0.059 | | 0.021 | | 0.549 | | 0.673 | | |
|  | human | 0.276 | 0.292 | 0.593 | 0.182 | 0.514 | | 1.000 | | 0.851 | | 0.788 | | |
|  | MB5 | 0.218 | <0.001 | 0.874 | 0.834 | 0.142 | | 0.805 | | 0.847 | | 0.580 | | |
| Human | chicken | 0.059 | 0.001 | 0.792 | 0.948 | 0.242 | | 0.141 | | 0.445 | | 0.705 | | |
|  | CO_2_ | 0.023 | <0.001 | 0.495 | 0.145 | 0.212 | | 0.021 | | 0.431 | | 0.875 | | |
|  | cow | 0.276 | 0.292 | 0.593 | 0.182 | 0.514 | | 1.000 | | 0.851 | | 0.788 | | |
|  | MB5 | 0.887 | <0.001 | 0.489 | 0.135 | 0.036 | | 0.805 | | 0.703 | | 0.768 | | |
| MB5 | chicken | 0.042 | <0.001 | 0.668 | 0.124 | 0.002 | | 0.085 | | 0.252 | | 0.937 | | |
|  | CO_2_ | 0.016 | <0.001 | 0.992 | 0.007 | 0.001 | | 0.010 | | 0.684 | | 0.890 | | |
|  | cow | 0.218 | <0.001 | 0.874 | 0.834 | 0.142 | | 0.805 | | 0.847 | | 0.580 | | |
|  | human | 0.887 | <0.001 | 0.489 | 0.135 | 0.036 | | 0.805 | | 0.703 | | 0.768 | | |

**Additional Table 8 Mean (±SE) of wild blood-fed mosquitoes caught outdoors using MM-X traps baited with natural and synthetic odour blends**

N=number of trapping nights

|  |  | *An. arabiensis* | *An. funestus* | *Culex spp.* | *Mansonia spp.* | *An. ziemmani* | *An. coustani* |
| --- | --- | --- | --- | --- | --- | --- | --- |
| Control | 25 | 1.40±0.56 | 0.04±0.04 | 1.20±0.56 | 0.32±0.15 | 0 | 0.20±0.12 |
| Cow | 25 | 2.24±0.83 | 0.32±0.13 | 1.56±0.59 | 0.40±0.173 | 0 | 0 |
| Chicken | 25 | 1.32±0.43 | 0.12±0.09 | 2.20±0.79 | 0.28±0.20 | 0 | 0.04±0.04 |
| Human | 25 | 1.76±0.58 | 0.32±0.14 | 1.68±0.42 | 0.36±0.21 | 0 | 0.08±0.56 |
| MB5 | 25 | 3.16±1.06 | 0.36±0.16 | 1.44±0.39 | 0.28±0.15 | 0 | 0.24±0.20 |

**References**

1. Guindon S, Dufayard J-F, Lefort V, Anisimova M, Hordijk W, Gascuel O: **New algorithms and methods to estimate maximum-likelihood phylogenies: assessing the performance of PhyML 3.0.** *Syst Biol* 2010, **59:**307-321.

2. Kearse M, Moir R, Wilson A, Stones-Havas S, Cheung M, Sturrock S, Buxton S, Cooper A, Markowitz S, Duran C: **Geneious Basic: an integrated and extendable desktop software platform for the organization and analysis of sequence data.** *Bioinformatics* 2012, **28:**1647-1649.
